# Cognitive potential of children with attention deficit and hyperactivity disorder

**DOI:** 10.5935/1808-8694.20130109

**Published:** 2015-10-08

**Authors:** Ana Carla Leite Romero, Simone Aparecida Capellini, Ana Claúdia Figueiredo Frizzo

**Affiliations:** a Speech and Hearing Therapist; MSc student in the Speech and Hearing Graduate Program - Philosophy and Sciences School - Paulista State University - FFC/UNESP - Marília - SP/Brazil.; b Senior Associate Professor of Written Language; Professor - Department of Speech and Hearing Therapy and Professor at the Graduate Program in Speech and Hearing Therapy - Philosophy and Sciences School - FFC/UNESP Marília - SP.; c PhD in Neurology; Professor - Department of Speech and Hearing Therapy and Graduate Program in Speech and Hearing Therapy - School of Philosophy and Sciences - Paulista State University - FFC - UNESP - Marília (SP), Brazil. Philosophy and Sciences School - Paulista State University - FFC/UNESP-Marília - SP/Brazil.

**Keywords:** attention deficit and hyperactivity disorder, auditory evoked potentials, electrophysiology

## Abstract

The literature has described comorbidities among the symptoms of children with Attention Deficit and Hyperactivity Disorder (ADHD) and the auditory processing changes, and these symptoms have been overlooked in the assessment, and consequently, on the rehabilitation of these individuals.

**Objective:**

To compare the findings of the long latency auditory evoked potentials in children with and without ADHD.

**Method:**

This is a historical cohort cross-sectional case-control study, in which we enrolled 30 children with and without ADHD, aged 8-12 years. We performed the long-latency auditory evoked potential test in two scanning procedures through passive and active tasks differing in frequency and duration (MMNf and MMNd) (P300f and P300D).

**Results:**

When comparing the performance of children with and without ADHD in the electrophysiological test assessment of hearing, we found significant differences concerning the P2 amplitude in the LE - which was higher for the ADHD group; and concerning the N2 amplitude and latency - which were abnormal in the ADHD group.

**Conclusion:**

This study provided a greater understanding of the central auditory pathways of children with and without ADHD when evaluated from electrophysiological tests.

## INTRODUCTION

Neuropsychological studies have reported that individuals with attention deficit and hyperactivity disorder (ADHD) have alterations in their prefrontal cortex and in subcortical structures, associated to frequent levels of inattention, impulsivity, hyperactivity, disorganization and social awkwardness, involving an inhibitory system deficit or in the working memory executive functions^1^, ^2^.

The literature has described comorbidities between symptoms of children with ADHD and auditory processing (AP) disorders, and such symptoms have been overlooked in the assessment and consequently the rehabilitation of these individuals^3^, ^4^.

Auditory processing disorder is common in children with ADHD and may be due to a deficit in the functioning of the auditory pathway, caused by changes in some of the central auditory nervous system (CANS) structures, or in the cerebral hemispheres, which can be observed by Long Latency Auditory Evoked Potentials (LLAEP), which assesses the auditory pathway integrity from the periphery all the way to the auditory cortex^4^.

According to the DSM-IV^5^, tests that require focused mental processing are abnormal in individuals with Attention Deficit and Hyperactivity Disorder when compared with control subjects, but it is not entirely clear which fundamental cognitive deficit is responsible for this.

Many studies have suggested that attention dysfunction is not the main cause of behavioral changes in individuals with ADHD^6^, ^7^ and the findings of evoked potentials showed that various sensory and cognitive stages are processed differently in individuals with ADHD, and this apparent discrepancy may be represented from studies of cognitive processes, by means of evoked potentials that examine the various brain regions^8^, ^9^.

Given the above, this study aimed to compare the findings of the auditory evoked potential latencies in children with and without Attention Deficit and Hyperactivity Disorder (ADHD).

## METHOD

This study project was submitted to the analysis and appreciation of the Ethics in Research Committee of this University and carried out after approval according to protocol number: 0094/2011. This historical cohort study was cross-sectional and of the case-control type.

The study included 30 children of both genders aged 8-12 years, divided into:

• Control Group (CG) - comprising 15 children with good academic performance, selected by the teachers following the criterion of satisfactory performance on two consecutive marking periods in a reading and writing assessment;
• Study Group (SG) - composed of 15 children properly diagnosed with ADHD by a multidisciplinary team, which included speech and hearing, neurological, educational, neuropsychological and educational assessments, considering the presence of at least six (or more) symptoms of inattention and six (or more) symptoms of hyperactivity-impulsivity persisting for at least six months, according to the Diagnostic Criteria for Attention Deficit/Hyperactivity Disorder from the DSM-IV. We employed Instruments from the neuropsychological battery of tests: WISC-III^10^ and the neuropsychological battery^11^. The children from the SG were assessed after a period of 24 hours without the use of medication (methylphenidate), since testing under the medication could mask the child's behavioral performance.

The children from both groups were evaluated after their guardians signed the Consent Form. All the children had chronological age between 8 and 12 years and were previously submitted to audiological, ophthalmological and psychological evaluations. Thus, we excluded from the study those individuals who did not have audiometric thresholds within the normal range (20 dB HL)^12^ and who had cognitive and visual acuity impairment.

Basic audiological evaluation was performed in a soundproof booth. For pure tone audiometry we used the GSI 61 (ANSI 3.6-1989 and S3.43-1992 standards) audiometer with TDH-50 phones. Hearing thresholds were obtained by air conduction, in the sound frequencies of 250-8,000 Hz. The normality criterion used was the classification proposed by Lloyd *&* Kaplan^12^, in which the average of the 500, 1,000 and 2,000 Hz frequencies should be equal to or less than 20 dB HL.

Evaluation of the Long Latency Auditory Evoked Potential was performed using the Biologic Navigator Pro and recorded with five disposable electrodes positioned at Fz and Cz in reference to the right (A2) and left (A1) lobes, using the two recording channels of the equipment, the ground electrode was placed on Fpz. Impedance was maintained at a level below 5 KW.

The components were surveyed in two sweeps, i.e., it was first elicited for tonal stimuli (tone burst) differing in frequency - MMNf and P300f (frequent stimulus: frequency of 750 Hz and rare stimulus: frequency of 1,000 Hz), and later, for stimuli differing in duration - MMNd and P300D (frequent stimulus: 100 ms and rare stimulus: 50 ms, both in the 1,000 Hz frequency).

Both stimuli differing in frequency and duration were randomly presented in an oddball paradigm, a rate of 1.1 stimuli per second, with a rare stimulus occurrence probability of 20% of the total 250 stimuli. The analysis time of the waves was 500 ms with a filter from 0.5 to 30 Hz and 50,000 mV sensitivity and alternating polarity.

For the MMN recording, the patient performed a passive task and was instructed to remain seated and relaxed but awake and watching a video (without sound) to get distracted and not pay attention to the sound stimulus presented to him. As for the P300 recording, the patient should undertake an active task, paying attention and discriminating the stimuli naming them as “thin” during P300f and “short” in P300d.

The stimulus presentation was randomized concerning the stimulated ear, alternating them to avoid result biases. Moreover, due to the difficulties inherent to the behavior of children with ADHD, we decided to replicate the test only when necessary, in routine use the Cz and Fz records, in order to verify and ensure data accuracy.

For final result analysis, we chose to use the records obtained at Cz, since in this study this was the region where the records were better; in addition, the literature has consistently described it as the region with the best visualization of these potentials.

In order to identify the P300 wave, we used the criterion proposed by Junqueira *&* Colafêmina^13^, which is presented below:

• Identification of the N1-P2-N2 complex - the first three waves that appear in the sequence and have negative - positive - negative polarity, respectively, occurring in the replication of the traces, frequent and rare, between 60 and 300 ms;
• P3 identification - the largest positive wave - immediately after the N1-P2-N2 complex, occurring in tracing replication for rare stimulus, between 240 and 700 ms;
• Latencies were marked on the highest peak, i.e. the point of maximum wave amplitude;
• The amplitudes were marked from the peak of the wave to the base line, and in the case of the N2-P3 inter-amplitude;

To identify the MMN waves, we considered the biggest wave of negative polarity, between latency values from 100-300 ms, viewed by the subtraction of the rare stimulus tracing from that of the frequent stimulus tracing^14^, ^15^. For the descriptive analysis of test results from the construction of tables with mean and standard deviation values per group and ear, we used the Shapiro-Wilk test to check for data normality. A comparison of the tests' mean values between the groups studied was made using the variance analysis - F test (ANOVA) and, when significance was found, it was confirmed by the Tukey test (ANOVA) - a parametric test that compares mean values using data variance, which necessarily constitute a normal distribution.

The result was described as *p*-value, and the level of significance adopted was always 5% or 0.05 (*p* ≤ .05).

## RESULTS

Upon assessing the electrophysiological evaluation of hearing during the active task with stimuli that varied on frequency, P300f, the right ear (RE) had statistically significant difference only in the N1 latency, while in the left ear (LE) there was no difference as to the level of significance in any of the variables.

Tables 1 and 2 show the mean values, standard deviation and *p*-value of the N1, P2, N2, P3 amplitude and latency values; and N2-P3 inter-amplitude in the P300f assessment in both groups of RE and LE, respectively.

**Table 1:** Descriptive statistics of the mean, standard deviation (SD) and *p*-value of the N1, P2, N2, P3 amplitude and latency variables and RE P300f N2-P3 inter-amplitude value.

Variable	Group	Mean	SD	*p*-value
Lat N1	CG	117.7	19.7	*0.0114^**^
	SG	99.4	17.1	

Amp N1	CG	-4.1	2.2	0.3699
	SG	-3.3	2.4	

Lat P2	CG	160.1	29.1	0.9682
	SG	159.7	36.5	

Amp P2	CG	-0.5	2.2	0.2462
	SG	0.5	2.7	

Lat N2	CG	207.4	31.5	0.7113
	SG	212.5	40.9	

Amp N2	CG	-5.8	2.6	0.3638
	SG	-5.0	2.2	

Lat P3	CG	316.0	32.2	0.6968
	SG	321.2	38.9	

Amp P3	CG	4.4	1.7	0.8271
	SG	4.6	3.2	

Int N2-P3	GC	10.5	4.2	0.6315
	SG	9.7	3.9	

Lat: Latency; Amp: Amplitude; Int: Inter-amplitude; SD: Standard Deviation, RE: Right Ear; Tukey Test ** Minimum Significant Difference = 13.84

**Table 2:** Descriptive statistics of the mean, standard deviation (SD) and *p*-value of the N1, P2, N2, P3 latency and amplitude variables and P300f N2-P3 inter-amplitude for the LE.

Variable	Group	Mean	SD	*p*-value
Lat N1	CG	112.8	25.1	0.5622
	SG	118.3	26.0	

Amp N1	CG	-4.5	3.6	0.2710
	SG	-3.1	2.9	

Lat P2	CG	155.6	35.9	0.4742
	SG	165.2	36.3	

Amp P2	CG	-1.4	3.2	0.0986
	SG	1.2	2.2	

Lat N2	CG	198.7	27.4	0.1284
	SG	219.5	43.5	

Amp N2	CG	-6.1	3.3	0.2435
	SG	-4.6	3.1	

Lat P3	CG	329.4	32.6	0.9329
	SG	328.5	29.8	

Amp P3	CG	5.2	4.4	0.7083
	SG	5.8	3.6	

Int N2-P3	CG	12.6	5.1	0.3919
	SG	11.0	4.8	

Lat: Latency; Amp: Amplitude; Int: Inter-amplitude; SD: Standard Deviation; LE: Left Ear.

In the P300 evaluation, when we stimulated with a stimulus that varied in duration, P300d, the RE had no statistically significant difference when the two groups were compared with and without ADHD; while for the LE it was significant when comparing the P2 and N2 amplitude and N2 latency.

Tables 3 and 4 show the mean value, standard deviation and *p*-value of the N1, P2, N2 and P3 latency and amplitude variables; and P300d N2-P3 inter-amplitude for the RE and LE, respectively.

**Table 3:** Descriptive statistics of the mean, standard deviation (SD) and *p*-value of the N1, P2, N2 and P3 amplitude and latency variables and P300d N2-P3 inter-amplitude for the RE.

Variable	Group	Mean	SD	*p*-value
Lat N1	CG	114.4	14.4	0.3985
	SG	107.0	29.9	

Amp N1	CG	-3.3	2.0	0.8291
	SG	-3.7	2.7	

Lat P2	CG	160.2	21.4	0.8283
	SG	162.9	41.4	

Amp P2	CG	0.8	2.2	0.6937
	SG	1.2	2.2	

Lat N2	CG	223.2	22.6	0.2994
	SG	234.5	34.1	

Amp N2	CG	-6.2	2.3	0.4603
	SG	-5.5	2.4	

Lat P3	CG	339.6	35.1	0.5325
	SG	331.3	36.9	

Amp P3	CG	3.9	2.6	0.2179
	SG	5.2	3.0	

IntN2-P3	CG	10.1	4.6	0.7740
	SG	9.7	3.9	

Lat: Latency; Amp: Amplitude; Int: Inter-amplitude; SD: Standard Deviation; RE: Right Ear.

**Table 4:** Descriptive statistics of the mean, standard deviation (SD) and *p*-value of the N1, P2, N2 and P3 latency and amplitude variables and P300d N2-P3 inter-amplitude for the LE.

Variable	Group	Mean	SD	*p*-value
Lat N1	CG	124.6	24.4	0.1926
	SG	111.3	30.2	

Amp N1	CG	-4.0	2.0	0.2610
	SG	-3.0	2.5	

Lat P2	CG	162.5	24.1	0.9701
	SG	163.0	35.0	

Amp P2	CG	-0.9	3.2	*0.0349^**^
	SG	1.3	2.4	

Lat N2	CG	208.7	21.7	*0.0213^**^
	SG	237.2	39.6	

Amp N2	CG	-6.6	2.7	*0.0203^**^
	SG	-4.4	2.2	

Lat P3	CG	331.7	28.8	0.5667
	SG	340.5	50.8	

Amp P3	CG	4.9	2.5	0.7665
	SG	4.6	2.9	

Int N2-P3	CG	11.5	3.6	0.0523
	SG	8.4	4.5	

Lat: Latency; Amp: Amplitude, Int: Inter-amplitude; SD: Standard Deviation; LE: Left Ear; Tukey Test ** Minimum Significant Difference: P2 Amp: 2.16; N2 Lat: 23.94 and N2 Amp: 1.88.

Comparing the electrophysiological evaluation of passive listening with stimulation with varied frequency, MMNf, both ears showed no difference as to the level of significance when the two groups - with and without ADHD were compared.

Tables 5 and 6 depict the mean, standard deviation and p-value of the N1, P2, N2 and P3 amplitude and latency variables; and MMNf N2-P3 inter-amplitude for the RE and LE, respectively.

**Table 5:** Descriptive statistics of the mean, standard deviation (SD) and *p*-value of the MMNf latency and amplitude variables for the RE.

Variable	Group	Mean	SD	*p*-value
Lat RE	CG	224.1	29.9	0.8019
	SG	220.2	52.6	

Amp RE	CG	-2.6	1.9	0.9948
	SG	-2.6	2.4	

Lat: Latency, Amp: Amplitude; RE: Right Ear; SD: Standard Deviation.

**Table 6:** Descriptive statistics of the mean, standard deviation (SD) and *p*-value of the MMNf amplitude and latency for the LE.

Variable	Group	Mean	SD	*p*-value
Lat LE	CG	224.1	29.9	0.2822
	SG	213.9	29.9	

Amp LE	CG	-3.4	2.0	0.9568
	SG	-3.4	2.2	

Lat: Latency; Amp: Amplitude; LE: Left Ear; SD: Standard Deviation; LE: Left Ear.

In assessing the MMN stimulation with varying stimulus for duration, MMNd, we did not find a statistically significant difference when comparing the two groups with and without ADHD in any of the variables in both ears.

Tables 7 and 8 depict the mean, standard deviation and p-value of the N1, P2, N2 and P3 amplitude and latency variables; and N2-P3 inter-amplitude ranging in MMNd duration for the RE and LE, respectively.

**Table 7:** Descriptive statistics of the mean, standard deviation (SD), minimum value, maximum value and *p*-value of the MMNd latency and amplitude variables of the RE.

Variable	Group	Mean	SD	*p*-value
Lat RE	CG	209.5	50.1	0.8407
	SG	206.1	41.3	

Amp LE	GC	-2.4	1.9	0.0881
	SG	-4.7	2.5	

Lat: Latency; Amp: Amplitude; RE: Right Ear, SD: Standard Deviation.

**Table 8:** Descriptive statistics of the mean, standard deviation (SD) and *p*-value of the MMNd latency and amplitude for the LE.

Variable	Group	Mean	SD	*p*-value
Lat LE	CG	245.4	57.7	0.4961
	SG	232.9	40.4	

Amp LE	CG	-3.7	2.6	0.9253
	SG	-3.6	2.6	

Lat: Latency; Amp: Amplitude; LE: Left Ear; SD: Standard Deviation.

## DISCUSSION

There are many studies that have evaluated the P300 in children with ADHD, but few have focused on the other LLAEP components: N1, P2 and N2^16^, ^17^. In this study, we found a statistically significant difference in the P300 latency and amplitude values between the CG and the SG; both in the P300f assessment as in the P300d, when concentrated on the N2, P2 and N2 components.

Concerning the P300d assessment, the LE had better P2 amplitude for the SG when compared to the CG, which corroborates studies^18^, ^19^ that reported that the P2 component is higher in children with ADHD when compared to normal controls.

The higher P2 amplitude in the SG children can be justified by studies^20^, ^21^ which claim that this wave has generators in various regions of the primary and secondary auditory cortex and reticular system, which are associated with the attention the subject pays to the sound stimulus and the inhibition of processing competitive stimuli; thus, children with ADHD in this study would require greater activation of these regions to ensure that they would remain vigilant and consequently discriminate rare stimuli from frequent ones.

In evaluating the P300d for the LE, we found a significant difference for the N2 amplitude, in which the CG had higher negativity when compared to the SG, and in N2 latency - where the SG values were more elongated. These results corroborate other studies^22^ and may be explained by possible pre-attentional and discriminatory difficulties in children with ADHD; since according to McPherson^23^ and Näätänen^24^ the N2 wave would happen from the attention and discrimination of a passive automatic pre-attentional response, elicited by discriminating the rare event.

With regard to N2 latency, the LE also showed a statistically significant difference when the SG was compared to the CG, and we found longer latency values for the SG, corroborating other studies^25^, ^26^ which analyzed, the N2 amplitude and latency values of children with ADHD, and compared with normal controls, finding an increase in N2 latency for the SG.

In this study, we observed that the N2 was the only component to show significant differences in terms of both latency and amplitude when children with ADHD were compared to those without it, which makes us consider that the ADHD children of our study had a decline in the efficiency of responses involving pre-attentional and discriminatory processes^19^, ^22^, since according to Näätänen^24^ N2 is generated from the attention and discrimination of a passive and pre-attentional automatic response elicited by the rare event discrimination.

Concerning the P3 component findings, it is consistent with studies^26^, ^27^, ^28^ which reported normal P300 latency and amplitude values in the ADHD group when compared to controls; however, these are discordant from other literatures, which have consistently described an increase in P300^16^, ^17^ latency time, as well as an amplitude decrease^8^, ^18^, ^19^ for individuals with ADHD.

A first explanation for not having significant differences between the P300 in children with and without ADHD in this study is that our sample was small, and this is a limiting factor vis-à-vis the specific discussion of these findings. Such a limitation in this study was described by Brayner^26^ and Satterfield & Braley^28^, who suggest a larger sample to better investigate the auditory pathway.

Another possible explanation is that children with ADHD have alterations in their pre-attentional and discriminatory processes - which was evident in the results found in the N2 wave; however, these children began to process this information in some way, which could be aided by other structures of the central nervous system and/or by interference of neural plasticity, in which sensory experiences bring about a better neural synchrony or a reorganization of the nerve cells^29^, since, according to the literature^30^ a series of cognitive processes may be involved in the P300 generation.

With regard to the MMN, both the MMNf and the MMNd did not show statistical difference for any of the variables, either in amplitude or latency when comparing the two groups. This suggests that children with ADHD in our study have deficits when they need to performa a discrimination task, while keeping sustained attention, or maintain attention for an extended period of time. To obtain the MMN, we need pre-attentional processes that are independent of the subject's response; thus, these children did not need to perform any task, which contributed to the normal MMN results found in the present study^31^, ^32^.

According to the specialized literature^24^, the MMN is elicited in the same way as the N2, or from attention and discriminatory activities of a passive and automatic pre-attentional response, elicited by the discrimination of a rare event. Moreover, N2 is recorded in the same region as the MMN^30^ latency and it has been commonly described as a functional representation of that component^33^, ^34^. Thus, in this study, we initially expected that children with ADHD would also have MMN alterations. One possible explanation for this result is that the ADHD subjects in this study still have deficits in sustained attention, since the most obvious LLAEP changes were seen as some kind of response was required, in which children would need to sustain attention for a long time while performing a task^32^.

In this study, we also found that the larger number of altered results came from the LE stimulation, leading us to suggest that, just like the processing of nonverbal stimuli, and the stimuli varying according to duration, are first processed by the right hemisphere^35^, ^36^ and transferred via the corpus callosum to the left hemisphere, changes in attention and discrimination of the children in this study may come from processing deficits in the right hemisphere.

## CONCLUSION

In comparing the performance of children with and without ADHD in the electrophysiological assessment of hearing, there were significant differences vis-à-vis the P2 amplitude in the LE, which was higher for the group with ADHD and for N2 amplitude and latency of the LE, which were abnormal in the ADHD group.

Regarding P300 and MMN, there were no significant differences when comparing both groups.

This study provided a greater understanding of the central auditory pathways of children with and without ADHD when evaluated with electrophysiological tests; however, further studies are needed, especially in the national literature, to better understand the functioning of the auditory pathway of these populations.
